# Current Advances in the Biosynthesis, Metabolism, and Transcriptional Regulation of α-Tomatine in Tomato

**DOI:** 10.3390/plants12183289

**Published:** 2023-09-17

**Authors:** Yuanyuan Liu, Hanru Hu, Rujia Yang, Zhujun Zhu, Kejun Cheng

**Affiliations:** 1 Collaborative Innovation Center for Efficient and Green Production of Agriculture in Mountainous Areas of Zhejiang Province, Key Laboratory of Quality and Safety Control for Subtropical Fruit and Vegetable, Ministry of Agriculture and Rural Affairs, College of Horticulture Science, Zhejiang A&F University, Hangzhou 311300, China; yyliu@zafu.edu.cn (Y.L.); 2020101031004@stu.zafu.edu.cn (H.H.); 201901070233@stu.zafu.edu.cn (R.Y.); 2 Chemical Biology Center, Lishui Institute of Agriculture and Forestry Sciences, Lishui 323000, China

**Keywords:** tomato, α-tomatine, biosynthesis, metabolism, transcriptional regulation, biological activity

## Abstract

Steroid glycoalkaloids (SGAs) are a class of cholesterol-derived metabolites commonly found in the *Solanaceae* plants. α-Tomatine, a well-known bitter-tasting compound, is the major SGA in tomato, accumulating extensively in all plant tissues, particularly in the leaves and immature green fruits. α-Tomatine exhibits diverse biological activities that contribute to plant defense against pathogens and herbivores, as well as conferring certain medicinal benefits for human health. This review summarizes the current knowledge on α-tomatine, including its molecular chemical structure, physical and chemical properties, biosynthetic and metabolic pathways, and transcriptional regulatory mechanisms. Moreover, potential future research directions and applications of α-tomatine are also discussed.

## 1. Introduction

Steroidal glycoalkaloids (SGAs), commonly known as *Solanum* alkaloids, are ubiquitously present in the *Solanaceae* plants, such as tomato (*Solanum lycopersicum*), eggplant (*Solanum melongena*), and potato (*Solanum tuberosum*) [1,2,3]. α-Tomatine, the predominant SGA in tomato, primarily accumulates within green tissues such as stems, leaves, and green fruits, and α-tomatine’s concentration dwindles in mature fruits [4]. Employed by plants as a natural defensive substance to combat various aggressors, including bacteria, fungi, viruses, insects, and herbivores, α-tomatine is considered an anti-nutritional substance because it poses toxicity risks for humans, potentially disrupting digestive functions and thereby impeding nutrient absorption [1,2,5]. Furthermore, α-tomatine’s bitterness can adversely affect the palatability of tomato and its processed products. As such, α-tomatine content remains a key determinant of tomato quality, holding significant importance for consumers. 

However, despite its toxicity, α-tomatine exhibits a plethora of physiological activities, encompassing anti-carcinogenic, anti-inflammatory, antioxidant, antifungal, antiviral, and antidiuretic effects [1]. Consequently, researchers have begun to appreciate α-tomatine’s potential value in medicine and health care, spurring its further investigation. 

As tomato fruits ripen, α-tomatine undergoes metabolic conversion to the non-toxic esculeoside A, another SGA, which also boasts a myriad of physiological benefits, such as inhibiting tumor growth, lowering cholesterol, and reducing the occurrence of atherosclerosis [6]. Therefore, elucidating the accumulation mechanisms of α-tomatine and its metabolites is crucial for enhancing the content of health-promoting compounds in tomato fruits.

## 2. Biosynthesis, Metabolism, and Transport of α-Tomatine

### 2.1. Biosynthesis of α-Tomatine

**Figure 1:**
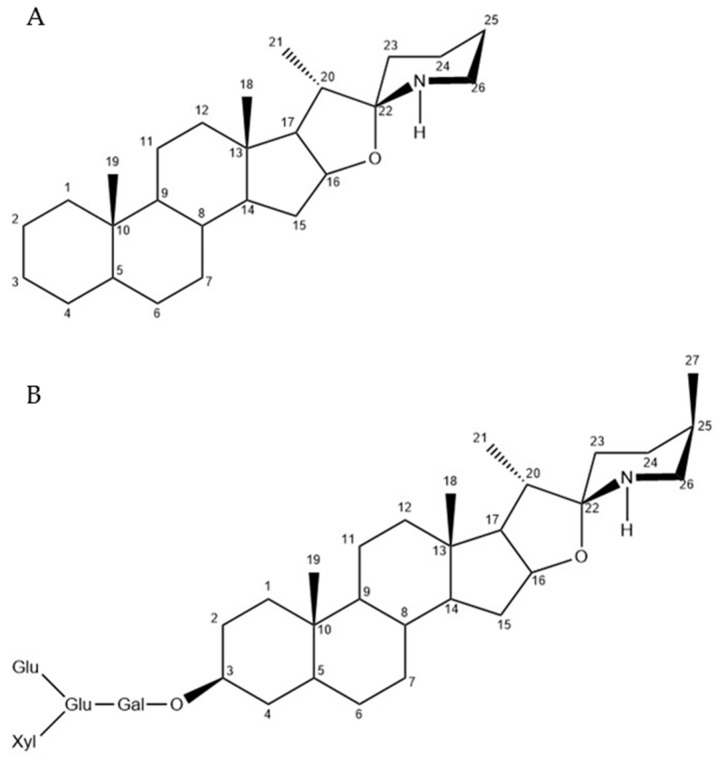
The structure of spirosolane (**A**) and α-tomatine (**B**).

**Figure 2:**
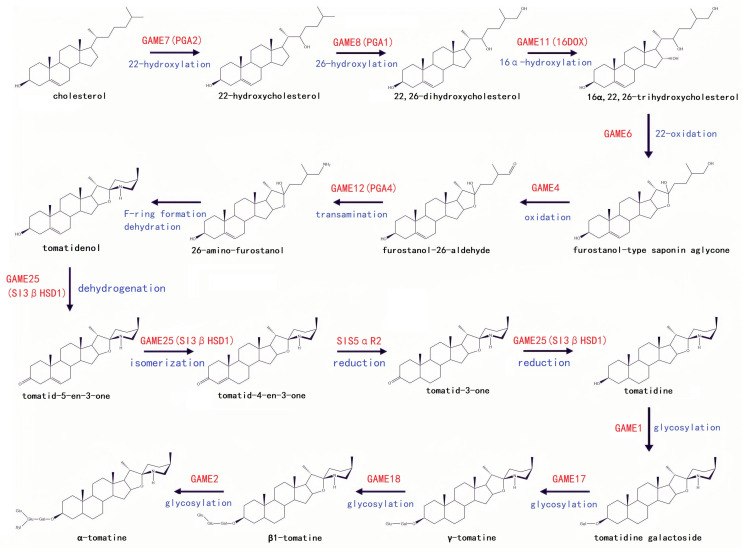
Putative biosynthetic pathway from cholesterol to α-tomatine. The black solid arrows indicate the single reaction step characterized previously. The names of the genes (enzymes) are depicted in red, and the enzymatic reactions are depicted in blue. 3βHSD, 3β-hydroxysteroid dehydrogenase; S5αR, steroid 5α-reductase.

α-Tomatine constitutes a C27 steroid, comprising a hydrophobic sapogenin (ligand) and a hydrophilic oligosaccharide chain. The sapogenin features a spirosolane-type aglycone tomatidine encompassing a nonpolar steroidal moiety and a polar nitrogen-containing heterocycle (Figure 1A), while the oligosaccharide chain, the branching structure of a tetrasaccharide, composed of one xylose molecule, two glucose molecules, and one galactose molecule, is affixed to the C-3 position of the aglycone tomatidine (Figure 1B) [7,8,9]. The biosynthetic and metabolic pathway of α-tomatine has been studied extensively, and the key genes have been elucidated (Table 1). α-tomatine’s biosynthesis commences with cholesterol, which undergoes a series of hydroxylation, oxidation, and transamination reactions to yield unsaturated steroidal alkaloid (SA) aglycones. Subsequently, SA is glycosylated to generate steroidal glycoalkaloids (SGAs) by different uridine 5′-diphosphate (UDP)–glycosyltransferases (UGTs) [1,10,11,12,13,14]. Specifically, cholesterol is first hydroxylated at the C-22 position by the cytochrome P450 family 72 (CYP72) monooxygenase (glycoalkaloid metabolism 7, GAME7) to form 22-hydroxycholesterol, which is then hydroxylated at the C-26 position by CYP72 monooxygenase (GAME8) to produce 22,26-dihydrocholesterol [15]. Following this, 22,26-dihydroxycholesterol undergoes further hydroxylation at the C-16α position by 2-oxoglutarate-dependent dioxygenase (GAME11) to form 16α,22,26-trihydrocholesterol [16]. Subsequently, 16α,22,26-trihydrocholesterol is subjected to oxidation and *E*-ring closure, mediated by CYP72 monooxygenase (GAME6), to yield furostanol-type aglycone [3]. The furostanol-intermediate is further oxidized by CYP88 family oxidase (GAME4) to produce 26-aldehyde furostanol [3], which then undergoes transamination catalyzed by transaminase (GAME12) to form 26-amino furostanol [17]. Following dehydration and *F*-ring formation, tomatidenol is generated (Figure 2).

Tomatidenol undergoes dehydrogenation, isomerization, and reduction reactions in the presence of short-chain dehydrogenase/reductase (GAME25) and steroid 5α-reductase (SlS5αR2) to form hydrolyzed tomatidine [18,19,20,21]. Hydrolyzed tomatidine is sequentially converted to tomatidine galactoside, γ-tomatine, β1-tomatine, and ultimately α-tomatine through the consecutive action of four glycosyltransferases (GAME1, GAME17, GAME18, and GAME2) [3] (Figure 2).

### 2.2. Metabolism and Transport of α-Tomatine

**Figure 3:**
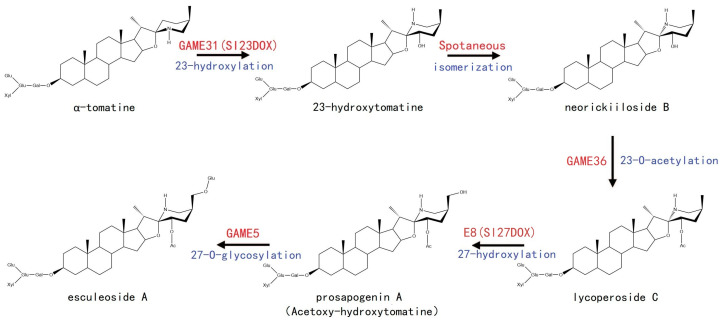
Putative metabolic pathway from α-tomatine to esculeoside A. The black solid arrows indicate the single reaction step characterized previously. The names of genes (enzymes) are depicted in red, and the enzymatic reactions are depicted in blue.

The content of α-tomatine is higher in immature green tomato fruits. As the fruit matures, α-tomatine is metabolized through hydroxylation, acetylation, and glycosylation to form neorickiioside B, lycoperoside C, and prosapogenin A, which are ultimately converted to the non-bitter and non-toxic esculeoside A. This results in the accumulation of higher levels of esculeoside A in red fruits [3,18,19,22]. As illustrated in Figure 3, α-tomatine is first hydroxylated at C-23 by α-tomatine 23-hydroxylase (Sl23DOX/GAME31) to produce 23-hydroxytomatine [23]. Subsequently, 23-hydroxytomatine is spontaneously isomerized to neorickiioside B [23]. Next, neorickiioside B undergoes acetylation at the C-23 hydroxy group by GAME36 to yield lycoperoside C (23-O-acetylated neorickiioside B) [24]. The lycoperoside C is then hydroxylated at C-27 by C-27 hydroxylase (E8/Sl27DOX) to form prosapogenin A (acetoxy-hydroxytomatine) [25]. Finally, prosapogenin A is glycosylated at the C-27 hydroxy group by glycosyltransferase (GAME5) to generate non-toxic and non-bitter esculeoside A [4,26]. Recent studies have indicated that α-tomatine is primarily stored in vacuoles, while its conversion to esculeoside A occurs in the cytosol. The GORKY transporter protein, located in the tonoplast membrane, is responsible for the transportation of α-tomatine from the vacuole to the cytosol, and facilitates the metabolic conversion of the bitter-tasting α-tomatine to non-bitter esculeoside A, rendering the fruit more palatable. Ripe *gorky* fruits still contain high levels of α-tomatine in the vacuoles, and no esculeosides are produced, thus exhibiting intense bitterness [27].

**Table 1:** Synthesis and metabolism genes of α-tomatine.

Gene ID	Gene Name	Other Name	Gene Function	References
Solyc07g062520	*GAME7*	PGA2/CYP72A188	Cytochrome P450 monooxygenase	[3]
Solyc06g061027	*GAME8*	PGA1/CYP72A208	Cytochrome P450 monooxygenase	[3,14,15]
Solyc07g043420	*GAME11*	Sl16DOX	2-Oxoglutarate-dependent dioxygenase	[16]
Solyc07g043460	*GAME6*	PGA2/CYP72A188	Cytochrome P450 monooxygenase	[3]
Solyc12g006460	*GAME4*	PGA3/CYP88D/SlCYP88B1	Cytochrome P450 monooxygenase	[3]
Solyc12g006470	*GAME12*	GABA-T2	γ-Aminobutyrate aminotransferase 2	[17]
Solyc01g073640	*GAME25*	Sl3βHSD1	3β-Hydroxysteroid dehydrogenases (3βHSD), 3-ketosteroid isomerase (3KSI), 3-ketosteroid reductase (3KSR)	[21]
Solyc10g086500	*SlS5αR2*		Steroid 5α-reductase	[20]
Solyc07g043490	*GAME1*		UDP-galactosyltransferase	[3]
Solyc07g043480	*GAME17*		UDP-glucosyltransferase	[3]
Solyc07g043500	*GAME18*		UDP-glucosyltransferase	[3]
Solyc07g043410	*GAME2*		UDP-xylosyltransferase	[3]
Solyc02g062460	*GAME31*	Sl23DOX	2-Oxoglutarate-dependent dioxygenase	[23]
Solyc08g075210	*GAME36*		BAHD acyltransferases	[24]
Solyc09g089580	*E8/Sl27DOX*		2-Oxoglutalate-dependent dioxygenase	[25]
Solyc08g006410	*GAME5*		UDP-glycosyltransferase	[26]

## 3. Transcriptional Regulation of α-Tomatine Biosynthesis and Metabolism

Currently, there are relatively few studies on the transcriptional regulatory mechanism of α-tomatine biosynthesis [14], with only a few regulatory factors having been identified (Table 2). Among these, *GAME9* is an AP2/ERF (APETALA2/ethylene response factor) transcription factor (TF). GAME9 can bind to the promoters of α-tomatine biosynthesis genes *GAME4* and *GAME7*, activating their transcription. GAME9 also regulates the expression of the *3-hydroxy-3-methylglutaryl CoA reductase 1* (*HMGR1*), *Δ(7)-sterol-C5(6)-desaturase* (*C5-SD*), and *sterol side chain reductase enzyme* (*SSR2*) genes. Overexpression of *GAME9* significantly increases α-tomatine content, while silencing *GAME9* significantly decreases α-tomatine content in tomato leaves and fruits [18,28]. Recent studies have shown that GAME9 also binds to the GC-rich element in the promoter of the glycosyltransferase gene *GAME17* during α-tomatine biosynthesis, activating the transcription of the *GAME17* gene. A 1 bp substitution in the AP2/ERF-binding domain of the GAME9 protein enhances its binding capacity to the *GAME17* promoter, which in turn increases α-tomatine biosynthesis [14]. 

SlMYC2 belongs to the basic helix–loop–helix (bHLH) type of TFs, which can activate the expression of *C5-SD* and *GAME4* alone, and can also coordinate with the AP2/ERF TF GAME9 to regulate the biosynthesis of SGA. Specifically, SlMYC2 and GAME9 act together on the promoters of the cholesterol synthesis gene (i.e., *C5-SD*), the mevalonate (MVA) pathway gene (i.e., *HMGR1*), and SGA core pathway genes (i.e., *GAME4* and *GAME7*) to synergistically activate their transcription in tomato plants [18]. Additionally, the bHLH-type TFs *SlMYC1* and *SlMYC2* have functional redundancy in regulating SGA biosynthesis [29]. SlMYC1 affects more α-tomatine biosynthesis-related genes compared to SlMYC2. In the *slmyc1* mutant, all α-tomatine and its precursor biosynthetic genes tested were down-regulated, but the regulatory mechanism of *SlMYC1* remains to be further elucidated [30].

*TOMATO AGAMOUS-LIKE 1* (*TAGL1*) is a member of the AGAMOUS branch of the MADS-box gene family. Using virus-induced gene silencing technology (VIGS) to silence the *TAGL1* gene, α-tomatine content and *GAME11* transcript levels were increased [31]. These results indicated that *TAGL1* gene silencing activated *GAME11* expression and promoted α-tomatine biosynthesis. On the other hand, silencing another MADS-box gene, *FUL1* (*FRUITFULL1*)/*TDR4,* using the VIGS technique also increased the α-tomatine content in tomato plants [32,33]. Further studies showed that both MADS-box TFs TAGL1 and FUL1, together with RIN (ripening inhibitor), form DNA-binding complexes, affecting the expression of *E8*/*Sl27DOX* genes and regulating the α-tomatine metabolic pathway [31,32,33].

In addition, phytochrome and cryptochrome proteins are involved in the interaction between the MVA pathway and the 2-C-methyl-D-erythritol 4-phosphate (MEP) pathway by positively regulating 1-deoxy-d-xylulose-5-phosphate synthase (DSX), affecting the biosynthesis of MEP and thus the synthesis of cholesterol, the precursor of α-tomatine. Photosynthesis has also been shown to feed the MEP pathway through the Calvin cycle [34]. SlHY5 (Elongated Hypocotyl 5) is a basic leucine zipper (bZIP) TF that integrates signals from photoreceptors and inhibits the activity of HMGR from the MVA pathway in tomato [35]. It has been shown that silencing *SlHY5* using the VIGS technique resulted in a significant down-regulation of the expression of the α-tomatine biosynthetic genes *GAME1*, *GAME2*, *GAME4*, *GAME11*, *GAME17*, and *GAME18*, and an increase in the expression of *GAME12* in tomato, ultimately leading to a decrease in α-tomatine content [36]. In contrast, silencing *SlPIF3* (*phytochrome interacting factor 3*), a member of the bHLH TF family [37], resulted in increased expression of *GAME1*, *GAME2*, *GAME4*, *GAME6*, *GAME11*, *GAME12*, *GAME17*, and *GAME18*, thus increasing the α-tomatine content [36]. Further studies have shown that SlHY5 and SlPIF3 regulate α-tomatine biosynthesis by binding to the G-box in the promoter sequences of *GAME1*, *GAME4*, and *GAME17*. In this process, SlHY5 plays a transcriptional activating role, while SlPIF3 plays a transcriptional repressive role [36].

The strictosidine synthase (STR-2), a member of the strictosidine synthase-like family, is up-regulated in tomato leaves infected with viruses, fungi, and bacteria. Overexpression of *STR-2* can increase α-tomatine content and enhance plant resistance to pathogens such as *Phytophthora infestans* and *Botrytis cinerea*, but its mechanism still needs further study [38]. Meanwhile, tomato microRNA1916 (miR1916) can target and regulate the *STR-2* gene, leading to cleavage of the *STR-2* transcript at the miRNA binding site, thereby inhibiting STR-2 enzyme biosynthesis. Therefore, the overexpression of *Solanum lycopersicum* (sly)-miR1916 reduces α-tomatine accumulation by decreasing the transcription of the *STR-2* gene [38].

## 4. Hormonal Regulation of α-Tomatine Biosynthesis and Metabolism

### 4.1. Regulation of α-Tomatine Biosynthesis and Metabolism by Jasmonic Acid

The phytohormone jasmonic acid (JA) plays a crucial role in the regulation of α-tomatine biosynthesis in tomato plants. The AP2/ERF TF *GAME9*, also known as *JRE4* (*Jasmonate-Responsive ERF 4*), is a key regulatory gene in the α-tomatine biosynthesis pathway. Studies have shown that the exogenous application of JA can induce the expression of *GAME9*/*JRE4,* as well as other α-tomatine biosynthetic genes, in tomato leaves and roots [39]. The effect of JA on α-tomatine biosynthesis is dependent on the JA receptor F-box protein CORONATINE INSENSITIVE 1 (COI1). JA can activate the expression of downstream *GAME9*/*JRE4* genes by binding to COI1, thus regulating the α-tomatine biosynthetic pathway [39].

Panda et al. (2022) pointed out that exogenous application of JA could increase the accumulation of α-tomatine in tomato plants, and the accumulation amount was positively correlated with the concentration of JA [30]. Inhibition of the JA biosynthesis gene *AOC* (*allene oxide cyclase*) by RNA interference (RNAi) technology decreased the α-tomatine content, and the gene expression of *GAME11* participating in core SGA biosynthesis pathway was significantly decreased. After exogenous application of JA, the α-tomatine content in *AOC-RNAi* transgenic tomato plants was partially recovered, and the expression of multiple genes was observed to be induced, including key genes in JA biosynthesis and signal transduction pathways (*jasmonic acid resistant 1* (*JAR1*), *COI1*, *JAZ1*, *JAZ2*, *JAZ6*, *MYC1* and *MYC2*), genes in SGA and sterol precursors (*SSR2*, *sterol methyltransferase 1* (*SMT1*)), core genes in the SGA biosynthetic pathway (*GAME4*, *GAME11* and *GAME12*), and transcriptional regulatory genes of SGA biosynthetic pathways (*GAME9*) [30]. Furthermore, the TFs SlMYC1 and SlMYC2 interacted with four JAZ proteins (JAZ1, JAZ2, JAZ6, and JAZ7) and are involved in JA signaling-mediated regulation of α-tomatine. In addition, SlMYC1 and SlMYC2 displayed partial redundancy in controlling the SGA pathway [30].

**Figure 4:**
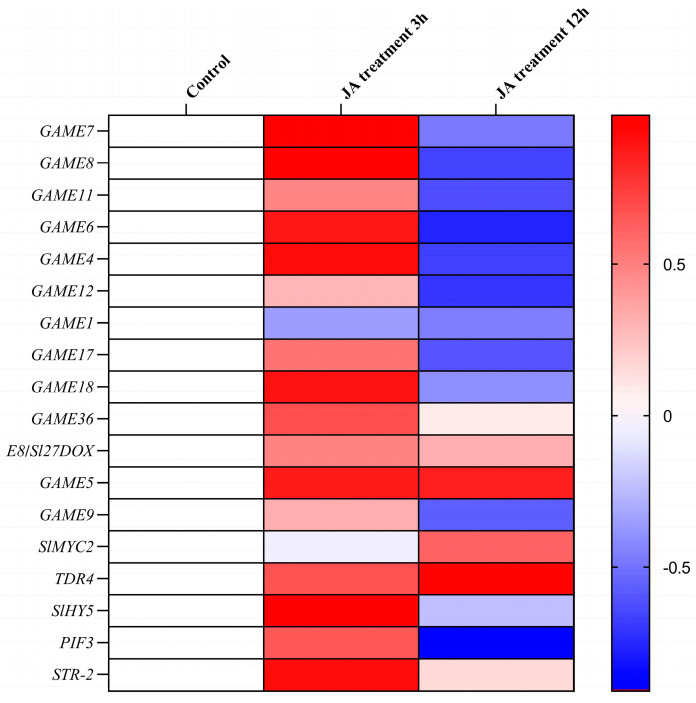
Transcriptome analyses of the effect of JA on the expression levels of α-tomatine biosynthesis- and metabolism-related genes. The average FPKM (for fragments per kilobase of exon per million fragments mapped) (log_2_ scale) of each gene is shown. Blocks with colors indicate decreased (blue) or increased (red) transcript levels relative to the corresponding control (two-week old tomato seedlings grown at the same time without 50 mM MeJA vapor treatments).

In order to further explore the effects of JA on α-tomatine biosynthesis and metabolism, transcriptome data of tomato leaves treated with JA were determined and analyzed in this study (Figure 4). The results showed that the expression levels of *GAME4*, *GAME6*, *GAME7*, *GAME8*, *GAME9*, *GAME11*, *GAME12*, *GAME17*, *GAME18* and *GAME36* were significantly increased at 3 h after JA treatment, and *E8*/*Sl27DOX*, *GAME5*, *SlMYC2*, *TDR4,* and *STR2* were significantly up-regulated at 3 and 12 h after JA treatment. Conversely, the expression of *GAME1* was inhibited by JA. These results suggest that JA can regulate the biosynthesis and metabolism of α-tomatine by influencing the expression of several key genes, highlighting the complex interplay between JA signaling and the regulation of α-tomatine content in tomato plants. 

### 4.2. Regulation of α-Tomatine by Interaction between JA and Gibberellin

Moreover, the interactions between JA and other phytohormones, such as gibberellins (GAs), can also impact the regulation of α-tomatine biosynthesis. These phytohormones can have synergistic or antagonistic effects on the expression of genes involved in the α-tomatine biosynthetic pathway. The study by Panda et al. (2022) provides evidence for the interplay between JA and GA signaling in the regulation of plant growth, SGA metabolism, and plant resistance to pathogenic fungi in tomatoes [30]. The changes in JA and GA content, which had an effect on plant growth, were coordinated with the SGA content in tomato. Both JA and the GA biosynthesis inhibitor polybulozole (PAC) inhibited the growth of tomato seedlings and induced the production of SGAs. The overexpression of *SlMYC2*, the core TF in the JA signal transduction pathway, increased the expression of GA catabolism genes (*GA2ox5* and *GA2ox7*) and decreased the level of active GA, suggesting that SlMYC2 could regulate the metabolism of GA at the transcriptional level. Similarly, SlMYC1 can also affect GA accumulation through the activation of *GA2ox5* and *GA2ox7* [30]. GA receptor GID1 is a key factor affecting α-tomatine accumulation. GID1-regulated DELLA proteins may promote the transcriptional activity of SlMYC1 and SlMYC2 by binding to JAZ proteins, thus regulating JA signal transduction and influencing the synthesis of α-tomatine mediated by JA signals [30]. The PAC-induced expression of genes like *JAZ6*, *SlMYC1*, *SSR2*, *GAME1*, *GAME11*, *GAME12*, and *GAME17* further supports the involvement of GA signaling in the regulation of α-tomatine biosynthesis. Overall, a complex signaling network that integrates JA and GA signaling to modulate α-tomatine synthesis could be achieved by the DELLA-mediated sequestration of JAZ proteins via interacting with JAZs to derepress MYC activity in order to activate JA signaling, or with JA-inducible MYC proteins in order to regulate GA metabolism (*GA2ox5* and *GA2ox7*) and signaling (*DELLA*). This, in turn, affects JA signaling to regulate SGA metabolism in tomato [30]. 

### 4.3. Ethylene Regulates the Conversion of α-Tomatine to Tomato Saponin A

Ethylene is a gaseous plant hormone that plays a crucial role in various aspects of plant growth, development, and response to environmental stimuli, including fruit ripening, senescence, and stress responses. The interplay between ethylene and α-tomatine biosynthesis and metabolism is an interesting aspect of plant hormone regulation.

Both ethylene and ripening can induce *E8*/*Sl27DOX* gene transcription and exhibit ethylene dose effects [40,41]. *RIN*, a key regulator of fruit ripening, can regulate ethylene synthesis and ethylene perception [42]. In addition, RIN has been shown to bind directly to promoters of *E8*/*Sl27DOX* [43,44,45]. Moreover, the ethylene response factor SlEFR.E2/4 could regulate the transcription of *E8*/*Sl27DOX* [46]. Iijima et al. (2009) found that exogenously applied ethylene could decrease the α-tomatine content and increase the esculeoside A content in mature green tomato fruits [6]. In ethylene synthesis-deficient mutant plants *rin* and *nor*, the lack of ethylene production leads to an accumulation of α-tomatine and a decrease in esculeoside A levels [6]. According to the metabolic pathway of α-tomatine, esculeoside A is formed from α-tomatine by two hydroxylations: one acetylation and one glycosylation. Through detecting putative intermediates [6], ethylene might act on the last step of α-tomatine conversion to esculeoside A, specifically the glycosylation at the O-27 position of prosapogenin A. Ethylene is likely to affect the glycosyltransferase gene *GAME5*, but no direct evidence to support this hypothesis has been reported yet. Further studies are needed in order to elucidate the specific molecular mechanisms by which ethylene interacts with the α-tomatine biosynthetic pathway and how these interactions influence α-tomatine biosynthesis and metabolism. 

**Table 2:** Transcriptional regulatory factors involved in the biosynthesis and metabolism of α-tomatine.

Gene ID	Gene Name	Other Name	Related Regulatory Pathway	Gene Family	References
Solyc01g090340	*GAME9*	*JRE4*; *Sl-ERF.B9*	Jasmonic acid, gibberellin	AP2/ERF	[28]
Solyc08g005050	*SlMYC1*	*JAMYC10*	Jasmonic acid, gibberellin	bHLH	[29]
Solyc08g076930	*SlMYC2*	*JA3*; *LEJA3*; *JAMYC2*; *LeMYC2*; *BHLH147*	Jasmonic acid, gibberellin	bHLH	[29]
Solyc07g042170	*JAZ1*	*TIFY10a*	Jasmonic acid, gibberellin	Jasmonate zim domain	[30]
Solyc12g009220	*JAZ2*	*TIFY10B*	Jasmonic acid, gibberellin	Jasmonate zim domain	[30]
Solyc01g005440	*JAZ6*	*TIFY11B*	Jasmonic acid, gibberellin	Jasmonate zim domain	[30]
Solyc11g011030	*JAZ7*	*TIFY5B*	Jasmonic acid, gibberellin	Jasmonate zim domain	[30]
Solyc08g061130	*SlHY5*	*thy5*	Light	bZIP	[36]
Solyc01g102300	*SlPIF3*		Light	bHLH	[36]
Solyc07g055740	*STR-2*		Sly-miR1916	Strictosidine synthetase	[38]
Solyc06g069430	*FUL1*	*TDR4*; *TM4*	Ethylene	MADS box	[33]
Solyc03g114830	*FUL2*	*MBP7*	Ethylene	MADS box	[33]
Solyc05g012020	*RIN*	*RIN-MC*; *MADS-RIN*; *LeMADS-RIN*	Ethylene	MADS box	[32]
Solyc07g055920	*TAGL1*	*ALQ*	Ethylene	MADS box	[31]
Solyc06g063070	*SlERF.E2*	*JERF1*	Ethylene	ERFs	[46]
Solyc01g065980	*SlERF.E4*	*SlERF6*	Ethylene	ERFs	[46]

## 5. A Probable Regulatory Network of α-Tomatine Biosynthetic and Metabolic Pathway

**Figure 5:**
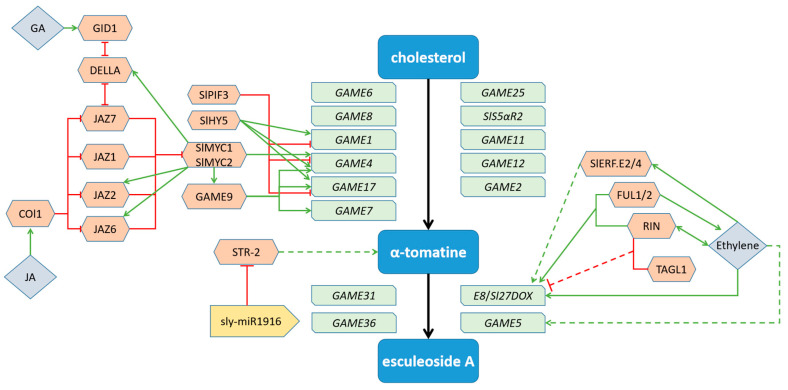
A model depicting the regulation of α-tomatine’s biosynthetic and metabolic pathways.

In summary, this paper proposes a model for the transcriptional regulation of α-tomatine biosynthetic and metabolic genes, as illustrated in Figure 5. The model suggests that α-tomatine biosynthesis is primarily regulated through the interplay of JA and GA: the JA receptor COI1 senses JA signals and mediates the degradation of downstream JAZ proteins, thus de-repressing the transcriptional activity of the SlMYC1 and SlMYC2 proteins. The SlMYC1/2 proteins and GAME9 protein collaboratively promote three downstream *GAME* genes (*GAME4*, *GAME7*, and *GAME17*), thereby increasing α-tomatine biosynthesis. GA mediates the degradation of the DELLA protein through the receptor GID1, thereby disassociating DELLA from JAZ proteins, which in turn interact with SlMYC2 and inhibit SlMYC2-regulated genes related to SGA biosynthesis and metabolism. The interplay between JA and GA signaling pathways in the biosynthesis of α-tomatine may serve as a mechanism by which plants can achieve a coordinated balance between growth and defense. JA, a well-established plant hormone, is known to elicit the biosynthesis of secondary metabolite α-tomatine, thereby enhancing plant defense against pathogens and herbivores. Conversely, GA is a classical phytohormone that promotes plant growth. The joint action of JA and GA in regulating the biosynthesis of α-tomatine is believed to facilitate the allocation of resources, striking a balance between the production of defensive specialized metabolites and growth processes. This coordination ensures that the costs associated with the production of defensive compounds do not impede the plant’s growth potential.

Additionally, light signaling also plays a role in regulating SGA biosynthesis. Light-responsive TFs SlHY5 and SlPIF3 enhance and reduce SGA biosynthesis through transcriptional activation and transcriptional repression of SGA biosynthesis genes (*GAME1*, *GAME4*, and *GAME17*), respectively [36]. Furthermore, the STR (strictosidine synthase-like) protein is a key enzyme involved in plant glycosyl alkaloid and terpenoid indole alkaloid synthesis pathways. sly-miR1916 was shown to play an essential role in the regulation of α-tomatine content, possibly through direct regulation of *STR-2* expression. Silencing of sly-miR1916 or overexpression of *STR-2*-enhances α-tomatine accumulation and disease resistance in tomato [38].

The regulation of α-tomatine metabolism is primarily achieved through the ethylene signaling pathway. The transition from α-tomatine to Esculeoside A during fruit ripening, which is regulated by ethylene signaling, represents a metabolic shift that may serve as a survival strategy employed by plants. In the vegetative growth stage and the early reproductive growth and development stage, α-tomatine functions as a defensive compound, aiding plants in resisting diseases and insect infestations. However, during the fruit ripening stage, α-tomatine undergoes metabolic transformations, leading to the formation of non-bitter and non-toxic Esculeoside A. This conversion enhances the palatability and desirability of tomatoes, facilitating their consumption by animals and promoting seed dispersal. The ethylene-dependent regulation of this process plays a crucial role in orchestrating the transition from α-tomatine to Esculeoside A. Ethylene signaling pathways are involved in the modulation of key enzymes and regulatory factors that mediate metabolic conversion, ensuring the timely and coordinated ripening of fruits. Specifically, ethylene acts as a signaling molecule to influence α-tomatine metabolism by regulating the expression of downstream genes (*E8*/*Sl27DOX* and *GAME5*). Among these, ethylene can induce the expression of the downstream TF *SlEFR.E2/4*, which in turn promotes the expression of the *E8*/*Sl27DOX* gene [46]. Moreover, the MADS-box TF RIN plays an essential role in regulating ethylene synthesis. RIN promotes ethylene production by interacting with ethylene signaling or binding to FUL1/2, thereby further augmenting the expression of *E8*/*Sl27DOX* genes [32]. Concurrently, RIN can also bind to TAGL1, forming a dimer that may impede the expression of *E8*/*Sl27DOX* genes. Additionally, ethylene may act on the glycosyltransferase gene *GAME5* to facilitate the conversion of prosapogenin A to Esculeoside A. However, the exact molecular mechanism requires further investigation in order for us to fully understand the complexities of the ethylene signaling pathway and its effects on α-tomatine metabolism. 

## 6. Concluding Remarks and Future Perspective

α-tomatine is a very important secondary metabolite in *Solanaceae*. Its biosynthesis and metabolism are completed by a series of genes and enzymes. Along with the different stages of plant growth and development, the biosynthesis and metabolism of α-tomatine is regulated by multiple transcriptional regulatory factors and hormonal pathways, including JA, GA, ethylene, light signals, and their downstream transcriptional regulatory factors. The synthetic and metabolic pathways of α-tomatine have been basically clarified, but the transcriptional and hormonal regulation that control the biosynthesis and metabolism of α-tomatine are relatively unclear. α-tomatine, as a defensive secondary metabolite, is induced by the defensive plant hormone JA and inhibited by the growth-promoting plant hormone GA in order to achieve balance between plant growth and defense. α-Tomatine is found mainly in green tissues, such as stems, leaves, and immature fruits. Along with fruit ripening, the content of α-tomatine reaches its highest point in the mature green stage, and then gradually metabolizes into the non-bitter, non-toxic Esculeoside A. The ripening-related transformation of α-tomatine is dependent on the ripening inducer plant hormone ethylene. When fruit is not ripe, the immature fruit is rich in α-tomatine, which is conducive to resisting diseases and pests and ensuring the survival of the plant. When the fruit is ripe, α-tomatine is degraded into non-bitter and non-toxic Esculeoside A, which makes the fruit delicious and conducive to feeding and seed spread. This mechanism might be regarded as a survival strategy for tomatoes.

In addition to survival strategies for the plant itself, α-tomatine also exhibits remarkable biological activities that make it an important compound for various applications, such as pest resistance and pharmaceutical applications. α-Tomatine possesses a broad range of pest resistance potential, making it an essential natural plant defense substance. In the context of promoting sustainable agriculture, using plant-derived pesticides like α-tomatine is a crucial research direction to minimize traditional pesticide use and protect the environment. Meanwhile, α-tomatine has shown promising results in the treatment of diseases such as cardiovascular diseases and cancer. However, it is essential to note that α-tomatine exhibits a certain degree of toxicity to humans, and its precise toxic mechanism of action remains elusive. Further research is needed to elucidate its toxic mechanism and discover ways to mitigate or inhibit α-tomatine’s toxicity, ultimately enhancing its applicability and value.

While significant progress has been made in elucidating the biosynthetic and metabolic pathways of α-tomatine, some key genes encoding enzymes involved in the synthesis process remain unidentified. In addition, compared to the study of key enzyme-encoding genes, the research on transcriptional regulation is relatively scarce, with reported transcriptional regulators primarily limited to a few *GAME* genes. Future research should focus on: identifying the missing key genes, completing the biosynthetic and metabolic pathways of α-tomatine, and identifying novel regulatory elements. Given the current extraction of α-tomatine, which is mainly from natural plants (low abundance and limited species), it is essential to deepen our understanding of its biosynthesis, metabolism, and transcriptional regulation mechanisms to facilitate the development of high α-tomatine germplasm using metabolic engineering approaches. 

In conclusion, the study of α-tomatine holds significant scientific and practical importance, and future research will supply a more profound and comprehensive understanding of its effects, providing critical scientific foundations and technical support for its applications in plant production and agriculture, as well as improved utilization in healthcare and disease treatment. This will ultimately maximize the potential of α-tomatine in various fields and contribute to the development of more sustainable and effective strategies for its production and use.

## Data Availability

The data presented in this study are available in this manuscript.
